# Supplementary material for: Occurrence of Clostridium perfringens in Wild Mammals in the Amazon Biome
Source: Animals (Basel). 2024 Apr 29;14(9):1333. doi: 10.3390/ani14091333 (PMC11083269; doi:10.3390/ani14091333)
Supplement: Supplementary file 1 [file animals-14-01333-s001.zip › animals-2951541-supplementary.pdf]

animals

MDPI

Communication

Occurrence of *Clostridium perfringens* in wild mammals in the Amazon biome

Hanna Gabriela da Silva Oliveira [1], Ananda Iara de Jesus Sousa [1], Isabela Paduá Zanon [2], Cinthia Távora de Albuquerque Lopes [1], Rodrigo Otavio Silveira Silva [2], Sheyla Farhayldes Souza Domingues [1] and Felipe Masiero Salvarani [1]*

[1] Instituto de Medicina Veterinária, Universidade Federal do Pará, Castanhal 68740970, PA, Brasil; hnnagabriela@gmail.com (H.G.d.S.O.); anandaiara@hotmail.com (A.I.d.J.S.); cinthia@ufpa.br (C.T.d.A.L.); shfarha@ufpa.br (S.F.S.D.); felipems@ufpa.br (F.M.S.);

[2] Laboratório de Bacterioses e Pesquisa da Escola de Veterinária, Universidade Federal de Minas Gerais, Belo Horizonte, MG, Brasil; pasuaisabela@gmail.com (I.P.Z.); rodrigo.otaviosilva@gmail.com (R.O.S.S.);

* Correspondence: felipems@ufpa.br

Supplementary Materials

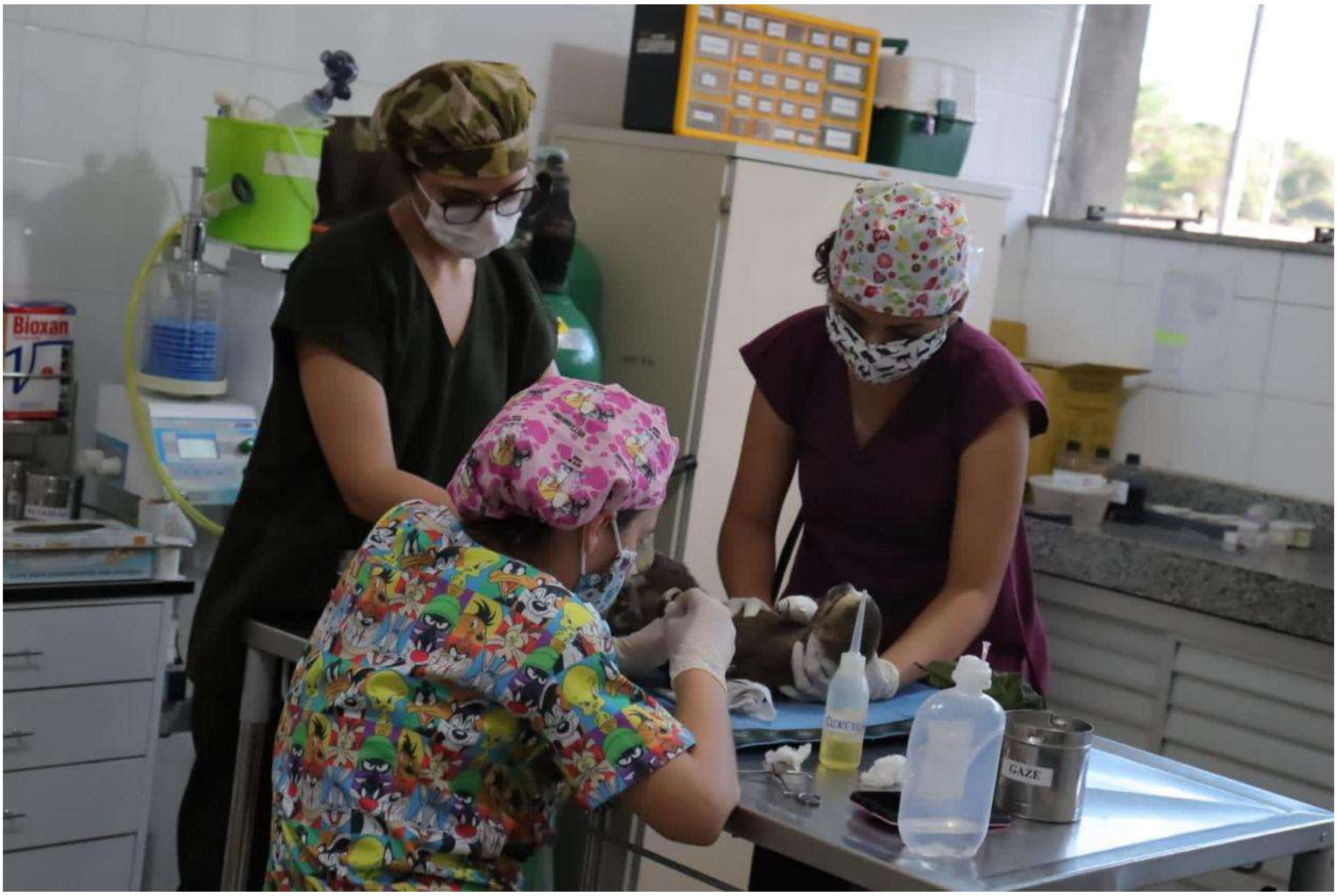

Figure S1. The collection procedures were monitored by veterinarian residents to monitor animal welfare, including the physical, mental and behavioural states of each species.

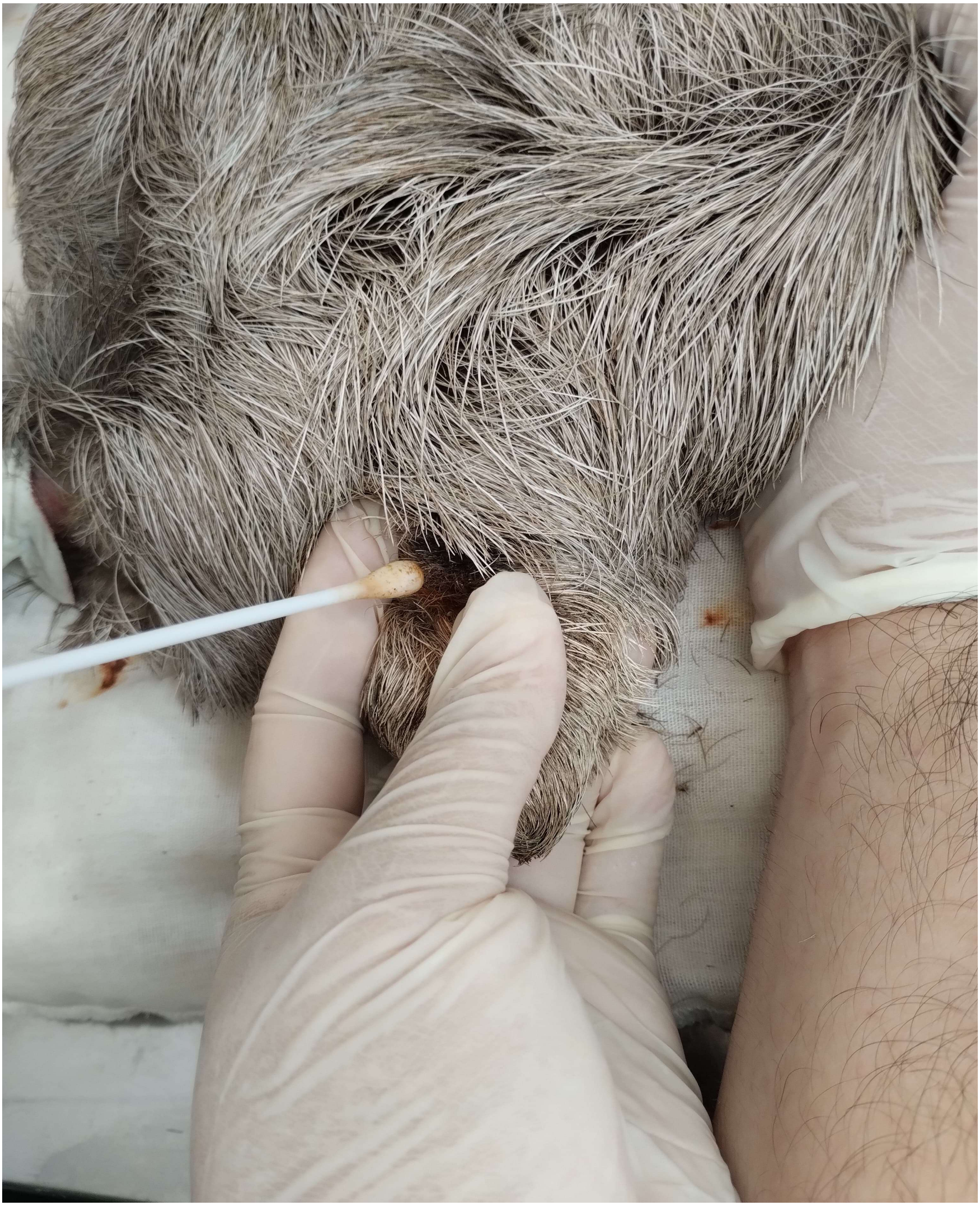

Figure S2. Cloacal and anal swabs were collected under the same conditions, and proper physical and chemical protocols for each species were applied to reduces the risk of accidents and stress.

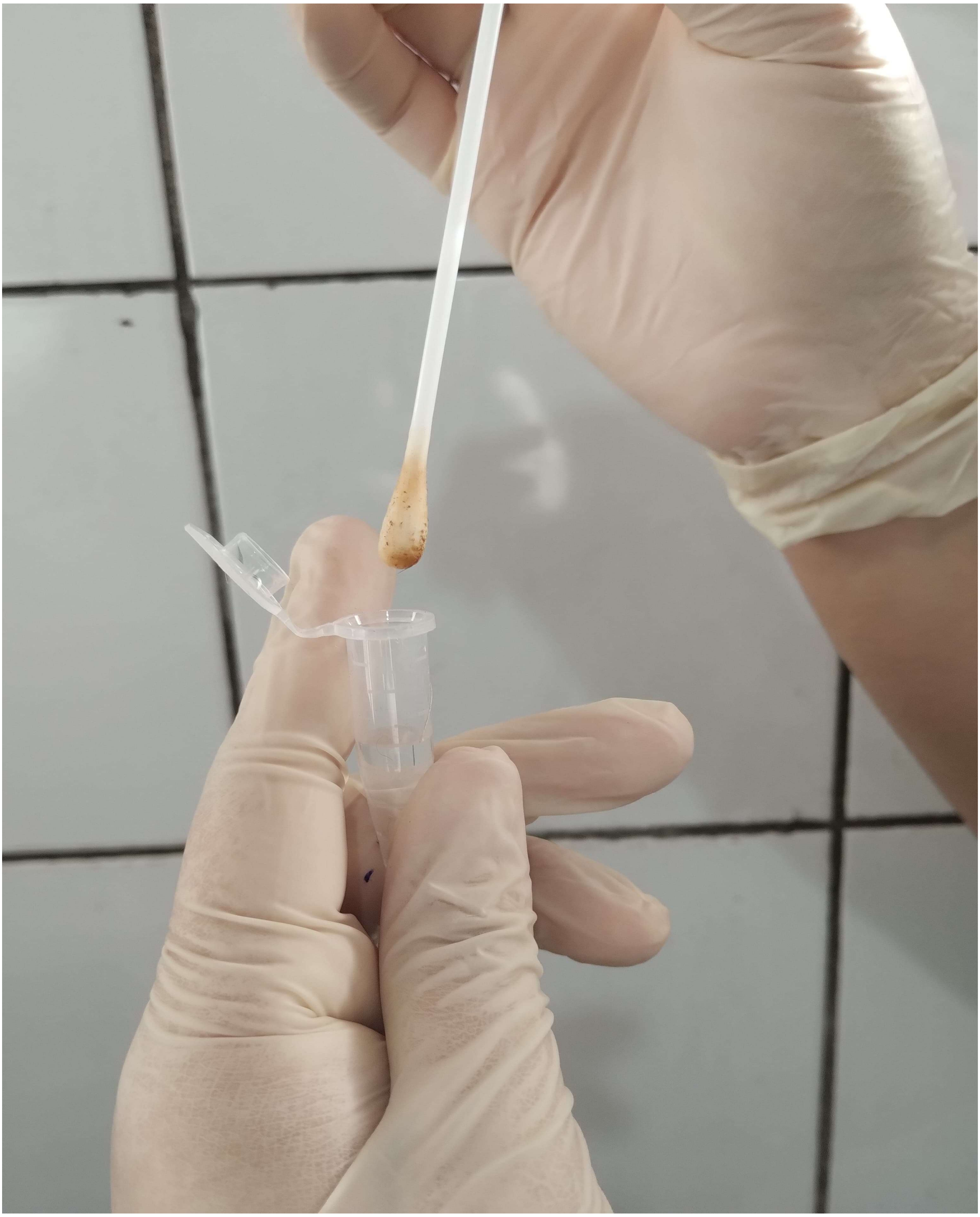

Figure S3. The stool samples were placed in microtubes and the swab samples were transferred to a saline solution.

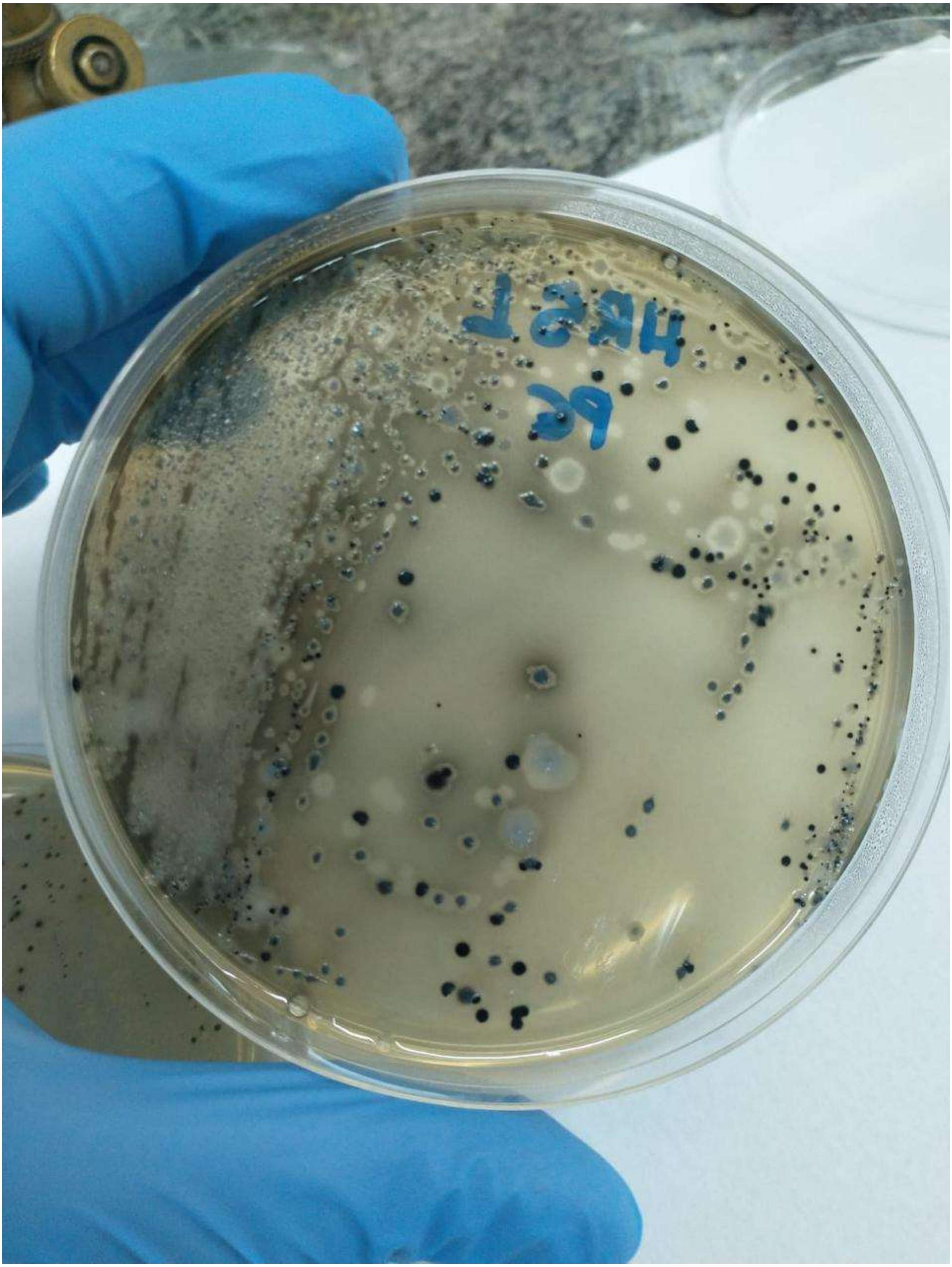

Figure S4. Characteristic colonies of *Clostridium perfringens* containing sulfite-reducing microorganisms.

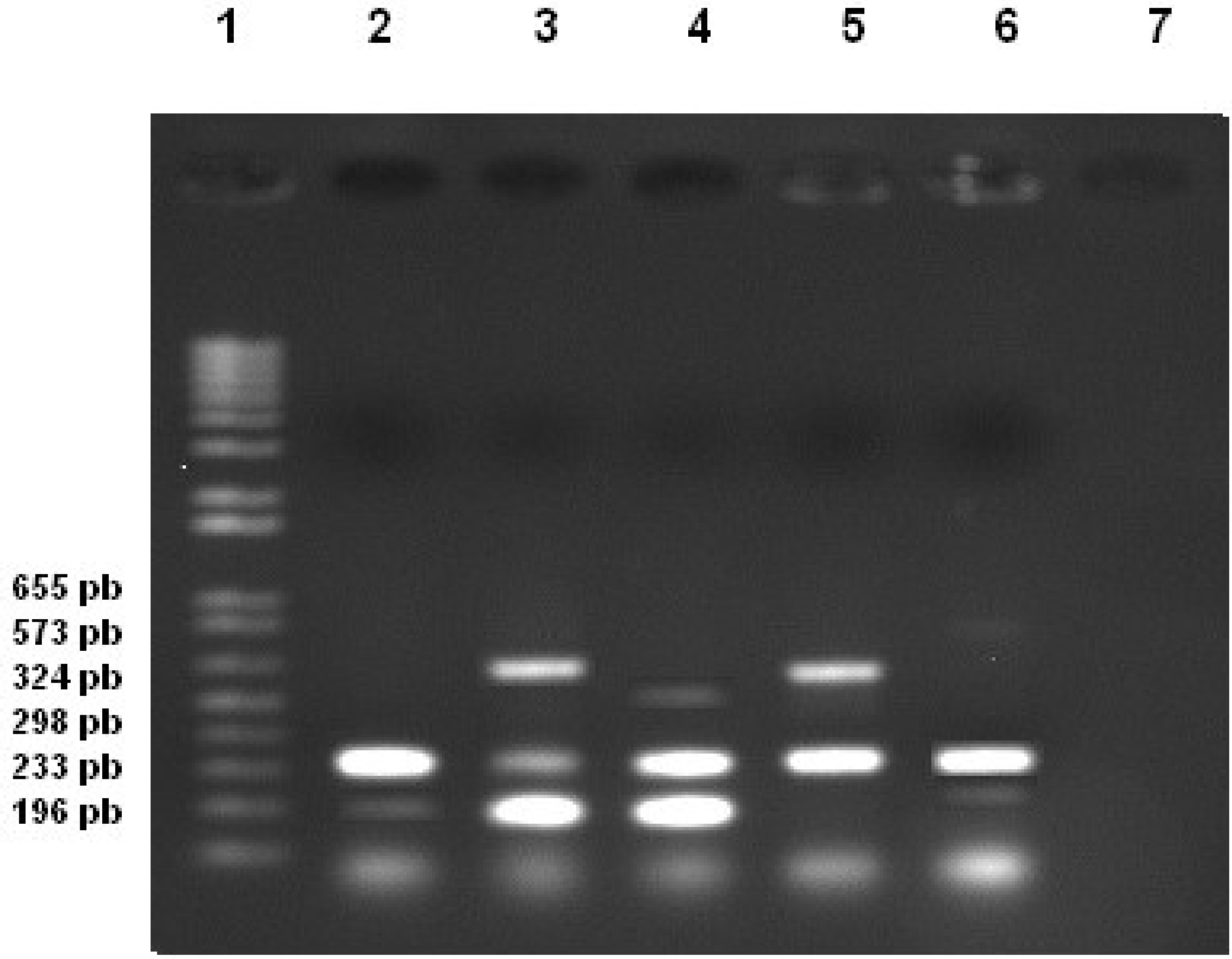

Figure S5. 1% agarose gel electrophoresis of the Multiplex PCR of *Clostridium perfringens* types A-E. Channel 1: Molecular size standard (1 kb plus DNA Ladder - Invitrogen); Channel 2- Amplification of the 233 and 324 base pair products of the *cpe* and *cpa* genes (*C. perfringens* A - ATCC 3624); Channel 3- Amplification of the 196 and 324 products of the *cpb*, *cpa* and *etx* genes (*C. perfringens* B - ATCC 3626); Channel 4- Amplification of the 196 and 324 products of the *cpb* and *cpa* genes (*C. perfringens C* - ATCC 3628); Channel 5- Amplification of the 324 and 655 base pair products of the *cpa* and *etx* genes (*C. perfringens D* - ATCC 3629); Channel 6- Amplification of the 298 and 324 base pair products of the *iA* and *cpa* genes (*C. perfringens E* - ATCC 27324); Channel 7 – Negative control.

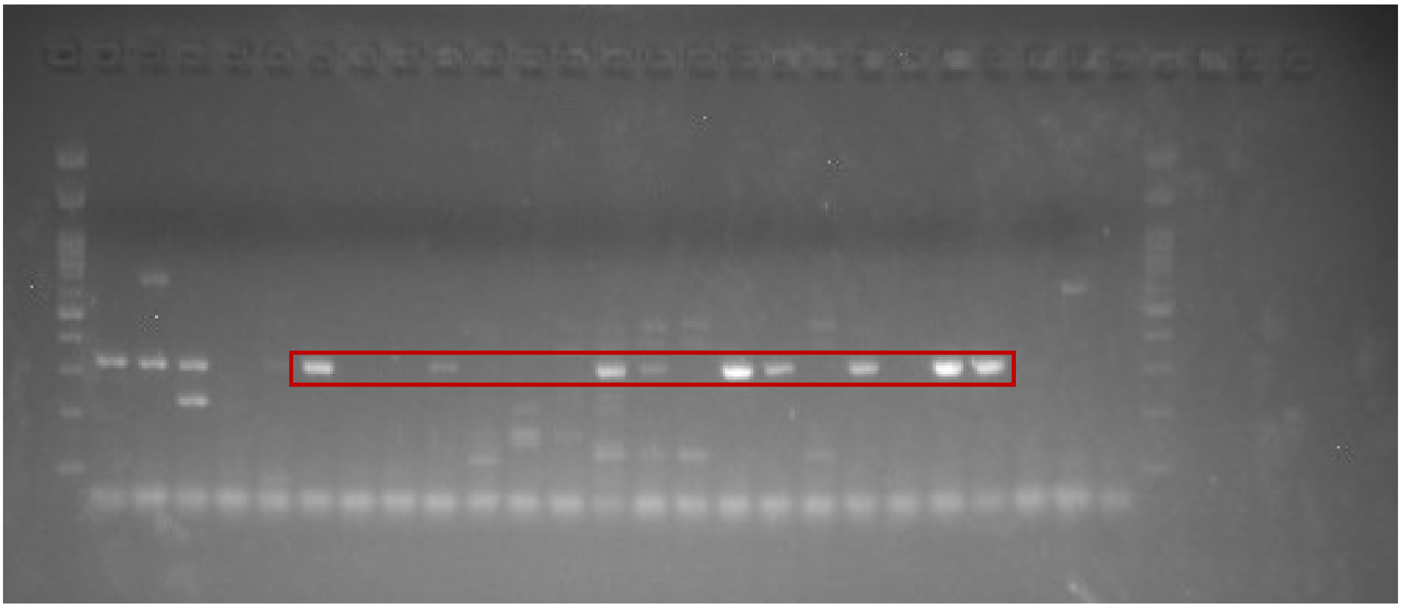

Figure S6. 1% agarose gel electrophoresis of the Multiplex PCR of *Clostridium perfringens* toxinotypes used in the study. Photo taken on the transilluminator equipment inside the exclusive PCR development room. Highlighted positive samples of *Clostridium perfringens* type A with amplification of the 324pb of the *cpa* gene.
